# Head-Eye movement control tests in patients with chronic neck pain; Inter-observer reliability and discriminative validity

**DOI:** 10.1186/1471-2474-15-16

**Published:** 2014-01-14

**Authors:** Eveline Della Casa, Jutta Affolter Helbling, André Meichtry, Hannu Luomajoki, Jan Kool

**Affiliations:** 1 Clinic for Manual Therapy, Zeltweg 83, 8032, Zurich, Switzerland; 2 Zurich University of Applied Sciences, School of Health Professions, Institute of Physiotherapy, 8400, Winterthur, Switzerland; 3 Provital Physiotherapy& GRAVITYtraining, Büelstrasse 29, 8132, Egg, Switzerland

**Keywords:** Head-eye movement control, Chronic neck pain, Whiplash associated disorders, Reliability, Discriminative validity, Assessment

## Abstract

**Background:**

Head-eye movement control deficit is an identified problem in patients with chronic neck pain, particularly in cases of whiplash associated disorders (WAD). To date, there is no evidence concerning the reliability and validity of visually assessed active head-eye movement control tests. Therefore, the objectives of the present cross-sectional study were, a) to develop a test battery; and b) to investigate inter-observer reliability and discriminative validity in patients with chronic neck pain compared to healthy controls.

**Methods:**

The study was conducted at two physiotherapy clinics in Switzerland. Ethics Committee approval was obtained. Ten active head-eye coordination tests, on 23 patients with chronic neck pain and associated symptoms and 19 healthy controls, were videotaped. The tests included eye movements in the neutral head position and 45° relative neck rotation, gaze stability and sequential head-eye movements. All tests were performed in the sitting and standing positions. Two blinded physiotherapists independently rated the randomized videos. Performance was rated as "negative", "moderately positive" or "clearly positive". Weighted kappa (wK) and 95% confidence intervals (CI) were calculated to investigate inter-observer reliability. Good reliability was defined as wK >0.5 with a lower boundary of 95% CI >0.2. Odds ratios (to define cut-off points) and the distribution of the classificator, numbers of positive tests, were calculated.

**Results:**

Three out of ten tests showed "excellent" (wK 0.82 to 0.86), five out of ten tests showed "substantial" (wK 0.69 to 0.79) and two out of ten tests showed "moderate" (wK 0.54 to 0.59) reliability. Results were comparable in the sitting and standing positions. On average, three out of five tests were rated positive in patients and one out of five tests was rated positive in healthy controls. An odds ratio of 13.3 to 18.6 was obtained using ≥2/5 tests as a cut-off point.

**Conclusion:**

Visual assessment by physiotherapists of head-eye movement control tests is reliable. The test battery is able to discriminate between patients with chronic neck pain and healthy controls. There were no differences in performance between the sitting and standing positions. The test battery can therefore be reduced to five tests. Further research is needed to identify the test-retest stability and responsiveness.

## Background

Neck pain is a common problem, with lifetime prevalence in the general population worldwide of up to 71% [1,2]. Impaired head-eye movement control is an identified problem in patients with chronic neck pain, particularly in patients with whiplash associated disorders (WAD). Symptoms associated with head-eye movement control impairment are dizziness, headache, light-headedness and visual disorders [3]. Movements requiring the coordination of neck, head and eyes are known to provoke those symptoms. A well-known example is driving a car after whiplash trauma, where Gimse found changed eye movements during car driving [4]. He also found altered eye movements during reading and differences in smooth pursuit eye movements with the head in the neutral position [5]. In patient anamnesis, symptom provocation was found during walking, fast head rotations and observation of moving objects. Oculomotor dysfunctions after cervical trauma were described by Hildingssons and colleagues [6-8]. Yahia and colleagues found abnormal dynamic and static balance in patients with chronic neck pain and dizziness [9]. Humphreys and colleagues [10] investigated neck pain in 180 patients: one third reported dizziness.

Following Treleaven and colleagues [3], dizziness is a frequent symptom in patients with neck pain and WAD. They assumed the underlying mechanism to be a convergence dysfunction of the sensorimotor input from the cervical spine, the vestibulum and the visual postural system. This theory is also supported by other authors [11-14]. Heikkilä et al. [11], found a reduced range of cervical motion and decreased upper cervical proprioception, which affects the voluntary eye movement. Peterson [13] postulated the head-neck motor system as an ideal model for understanding issues of complex motor control and Armstrong [14], mentioned that sensory information from neck proprioceptors is processed in tandem with information from the vestibulum, cerebellum and cortex.

Following diagnosis of a dysfunction of convergence, the management of an intervention is based on the clinical presentation and the functional impairment. The following assessments are recommended to measure the functional impairment and are used in clinical practice: joint position error (JPE) [12], "The Fly" movement method [15], smooth pursuit neck torsion test (SPNT) [16], postural stability and balance, as well as oculomotory and head-eye coordination tests [17,18].

In this study, the selection of our tests was influenced by some of the research tests outlined above. The test battery had to meet the following criteria: have the ability to transfer the research results into practice; be simple; performed without high-tech equipment; easy to learn; and, preferably, be low cost. The test battery of five tests was performed in the sitting position and the standing position. The latter, with feet together to achieve a more challenging balance position [19].

Eye movement was tested according to Jull and colleagues [19]. They tested eye-follow in both neutral and in relative 45°neck rotation, using the elements of the smooth pursuit neck torsion test from Tjell [16]. Tjell compared eye movements performed with the head in neutral position to a position in 45° relative head rotation. He reported significant differences between these two positions in patients with whiplash trauma compared to patients with vestibular pathology. Because of the unavailablity of equipment for standardizing eye movement velocity, in the present study the eye movement tests were performed differently to Tjell et al. [16]. The participants were asked to move their eyes horizontally from side to side as quickly as possible, while actively maintaining their initial fixed head position.

Treleaven and colleagues [3] showed that patients with WAD have decreased velocity of movement in the sequential head and eye movement test and in the gaze stability task, compared to healthy controls. Furthermore, patients with WAD have a reduced active range of motion of the cervical spine in the gaze stability test. These altered movement patterns were associated with a higher score in the Neck Disability Index (NDI) [3]. However, this data was collected using sophisticated laboratory equipment to measure eye movement in association with head movement. A physiotherapeutic diagnosis, is based on visual observation of the patients’ impaired head-eye movement. According to Treleaven [20] visual clinical observation of the quality of head and eye movement, in conjunction with symptom provocation, is the suggested method for assessment of oculomotory dysfunction. Qualitative and quantitative aspects of movement, as well as symptom provocation, are references for physiotherapeutic intervention [10,19]. Range of motion and velocity of movement is difficult to measure without technical equipment but the quality of movement control can be visually inferred [21]. To date, the reliability of visual observation of head-eye coordination tests has not been investigated.

The main objectives of the present study were to:

a) Develop a test battery

b) Investigate the inter-tester reliability of the visual observation of ten videotaped head-eye coordination tests

c) Determine the discriminative validity of these tests in patients with chronic neck pain compared to healthy controls

Our hypothesis was that, "Visual assessments by physiotherapists of head-eye movement control deficit are reliable and that these tests are able to discriminate between controls and patients".

We also hypothesized a greater head-eye control deficit in the standing position compared to sitting, since the latter is a more challenging balance position and requires increased coordination.

## Methods

### Design

A cross-sectional study, conforming to the Declaration of Helsinki, was performed at two physiotherapy outpatient clinics in Switzerland. Ethical approval was obtained from the Ethics Committee of Canton Zurich. Written informed consent was provided by all participating subjects, as well as for permission to use the photographs in the manuscript. Ten head-eye movement control tests were videotaped from a face-front perspective, with five performed in the sitting and five in the standing position [19]. Two experienced (OMT IFOMPT qualified) blinded physiotherapists independently rated the randomized videos.

### Participants

Based on a previous study by Treleaven et al. [3], a sample size of 40 participants was used in this study. All patients were recruited over a 2-month period and had been referred to the clinics either by a general practitioner, chiropractor or other physiotherapist. During the same period, healthy controls were recruited from a training centre and from our circle of acquaintances.

Males and females aged between 25 and 70 years were included (Table 1). Patients were required to have suffered from non-specific chronic neck pain during the last 3 months, an NDI score of >10/100 [22,23] and a minimum of one symptom associated with head-eye movement control impairment, such as dizziness, light-headedness or a visual disorder.

**Table 1:** Characteristics of participants

**Group**	**Patients**	**Controls**	**Total**
Number	23	19	42
Female/Male	23 / 0	12 / 7	35 / 7
Age: mean / min.-max.	50.7 / 29–70 years	50.9 / 31–70 years	
NDI mean / min.-max.	32.2 / 10-62	3.3 / 0-8	
Neck Trauma yes / no	17 / 6	2 / 17	

•Neck disability index score NDI 0–100 points.

•Age (p-value) = 0.939.

The NDI is a reliable and valid instrument in the assessment of the limiting factors in patients with chronic neck pain [22-24]. (Scoring interpretation for the NDI [23]: <10/100 = none, 10-28/100 = mild, 30-48/100 = moderate, 50-68/100 = severe, >70/100 = complete).

For inclusion, patients had to have a minimum of 45° active range of motion rotation to both sides and be able to stand freely with feet together without risk of falling. Included in the study were 17 patients with neck trauma and 6 (26%) without trauma.

Excluded from the study were subjects with known eye diseases, specifically blindness, strabismus, nystagmus, trochlear nerve injury, central nervous system disease and vestibular disease (such as Menière’s disease). Furthermore, a history of ear surgery, dizziness caused by vascular problems and neck pain caused by cervical radiculopathy, were grounds for exclusion.

Healthy controls were excluded if they had reported visual disturbances, dizziness or neck pain during the last six months. Two subjects in the control group reported minor historical neck trauma, but had not experienced any symptoms for many years.

An age-matched control group was recruited because there is evidence to show that age affects head-eye coordination [25,26].

### Standardized testing procedure

The order of the tests was standardized:

1) Eye movements neutral; 2) Gaze stability; 3) Sequential head and eye movements; 4) Eye movements in 45° relative neck rotation to the right; and 5) Eye movements in 45° relative neck rotation to the left. These five tests were performed in the sitting position and then repeated in the standing with feet together position. The markers and camera were placed at participants’ eye level. The participants were given verbal instructions and were asked to move as precisely and as quickly as possible during the tests. Each test was recorded on video for 10 seconds. No resting time was allowed between tests, except to allow a short instruction time for the following test. In the case of symptom provocation, subjects were advised to stop. Spectacles and contact lenses were worn as usual.

### Test facility

**Figure 1:**
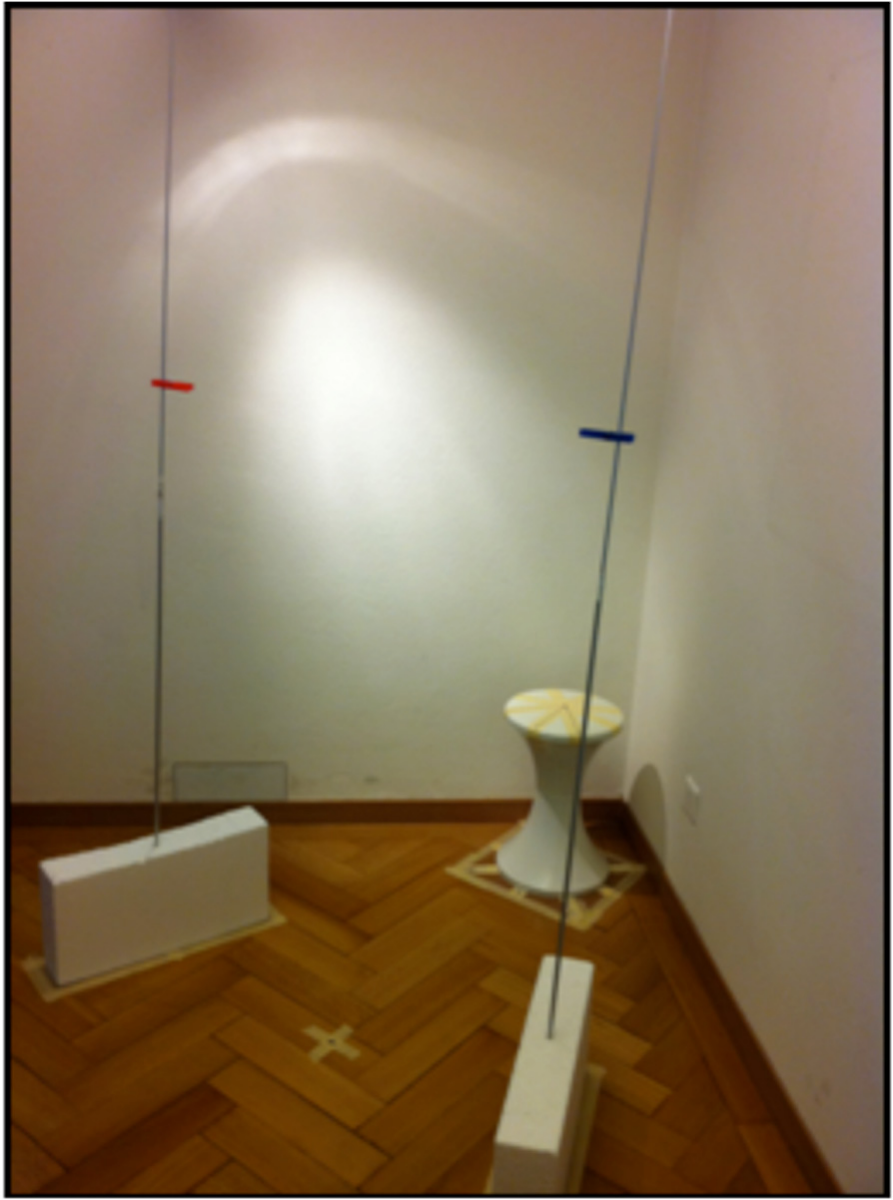
**Test facility for sitting posture. Blue and red markers are at participants’ eye level.** The cross on the floor marks the camera position (also at eye level).

**Figure 2:**
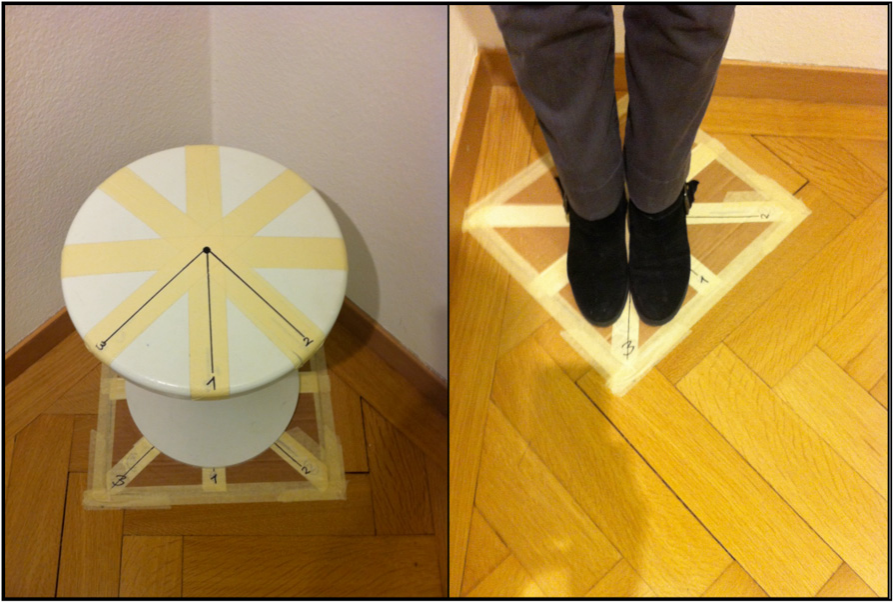
Three arrows mark the starting positions in sitting (left) and standing (right) for the tests with neutral head position (Position 1) or 45° right/left relative neck rotation (Positions 2 and 3).

To prevent patient falls, the test facility (Figures 1 and 2) was situated in a corner of the room. An equilateral triangle, with sides of 1 m in length, was marked on the floor. A chair and a footprint were positioned at one side of the triangle and two sticks in styrofoam sockets at the other sides. Both sticks had coloured markers whitch could be adjusted to participants’ eye level. The camera (type: Canon Legria HF R205) was positioned on a tripod exactly in the centre of the triangle and could also be adjusted to eye level. The angle between the camera and one side of the triangle amounted to 30°, resulting in a standardized gaze change of 30° to both sides.

Three arrows were marked on the chair for the start of the sitting tests and three on the footprint for the standing tests. Position 1 was placed exactly in front of the camera and marked the neutral head position. Position 2 was placed at an angle of 45° to the right of the camera (Position 1) and Position 3 was placed 45° to the left of the camera. For the tests in 45° relative neck rotation to the right, the participants sat or stood on position 3 and turned their head towards the camera, thus creating the 45° relative neck rotation to the right.

### Tests

#### Eye movements

**Figure 3:**
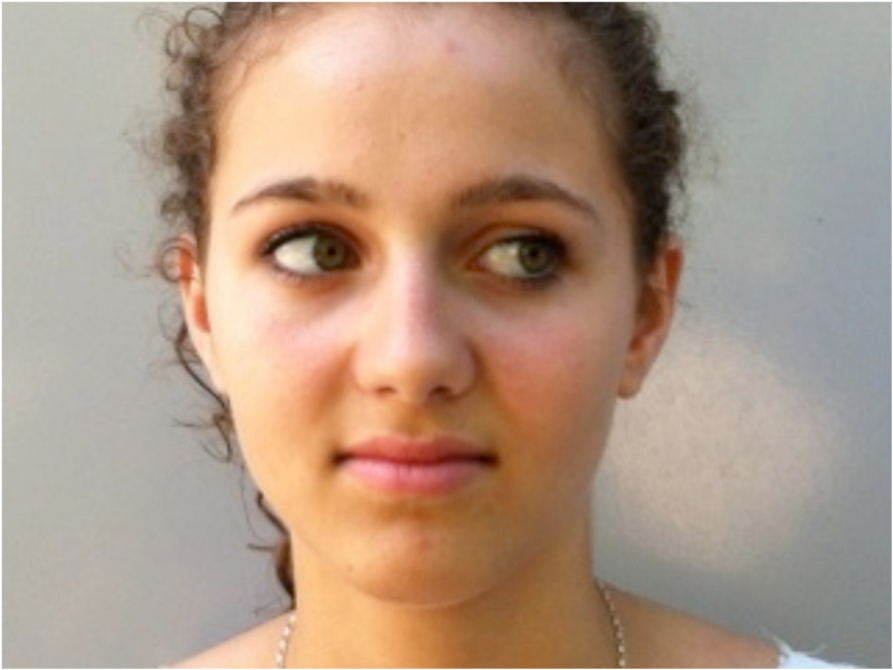
Eye movements to right and left while maintaining the head in the neutral position.

Eye movements (Figure 3) were performed according to Jull and colleagues [19].

#### Procedure

Test in the sitting position: participants sat on the chair with an upright posture at position 1. The markers were adjusted to eye level.

Test in the standing position: The participants stood on the footprint with feet together at position 1. The markers were adjusted to eye level.

The subjects were required to maintain their head in the neutral position while moving their eyes sideways from one marker to the other as fast as possible for 10 seconds.

Instruction: "Keep your head in the previously adjusted neutral position while moving your eyes between the two markers as fast as possible".

The test rating scale is shown in Table 2.

**Table 2:** Rating scale of eye movements in neutral head position

**Rating**	**Definition**
0 = negative	Smooth, precise, fast eye movements, fast change of gaze direction, head remains stable
1 = moderately positive	Slightly irregular eye movements. Short stops before changing gaze direction. Head slightly unstable
2 = strongly positive	Eye movements clearly slower or irregular. Prolonged maintenance of gaze direction before changing direction. Obvious head movement. Test cannot be performed

#### Gaze stability

**Figure 4:**
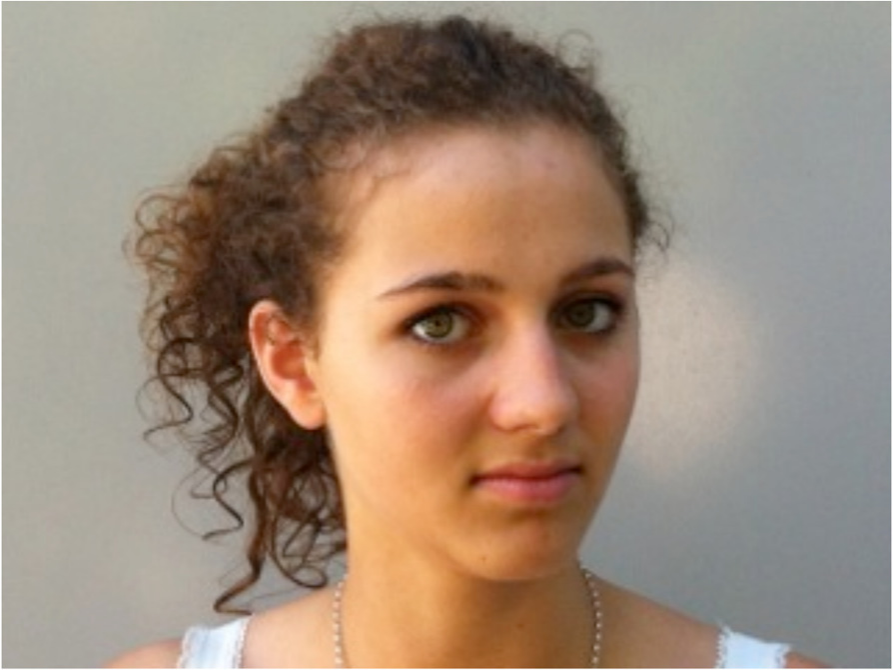
Gaze Stability.

The test is shown in Figure 4 and the rating scale in Table 3.

**Table 3:** Rating scale of gaze stability

**Rating**	**Definition**
0 = negative	Gaze stable. Smooth, well-coordinated, precise and fast movement of the head, fluent change of head movement direction
1 = moderate positive	Gaze stable. Slightly irregular head movement
2 = strong positive	Gaze repeatedly unstable. Head movement slow and irregular

#### Procedure

Test in the sitting position: participants sat on the chair with an upright posture at Position 1. The camera was adjusted to eye level.

Test in the standing position: participants stood on the footprint with feet together at Position 1. The camera was adjusted to eye level.

The subjects were asked to hold a stable gaze on the camera while moving their head to left and to right as far and as fast as possible for 10 seconds.

Instruction: "Hold your gaze stable on the camera while moving your head left and right as far and as fast as possible".

#### Sequential head and eye movements

Dissociated sequential head and eye movements according to Treleaven and colleagues [3] were performed in a range of motion of 30° to both sides.

#### Procedure

Test in the sitting position: participants sat on the chair with upright posture at Position 1. The markers were adjusted to eye level.

Test in the standing position: participants stood on the footprint with feet together on position 1. The markers were adjusted to eye level.

Starting in the neutral position, the participants were to move their eyes to the right marker with the head remaining still. Subsequently, they were to rotate the head in the same direction with gaze remaining fixed. With the head facing the right marker, the eyes were then to focus on the left marker, followed by the leftwards rotation of the head with fixed gaze. These movements were to be repeated for 10 seconds.

**Figure 5:**
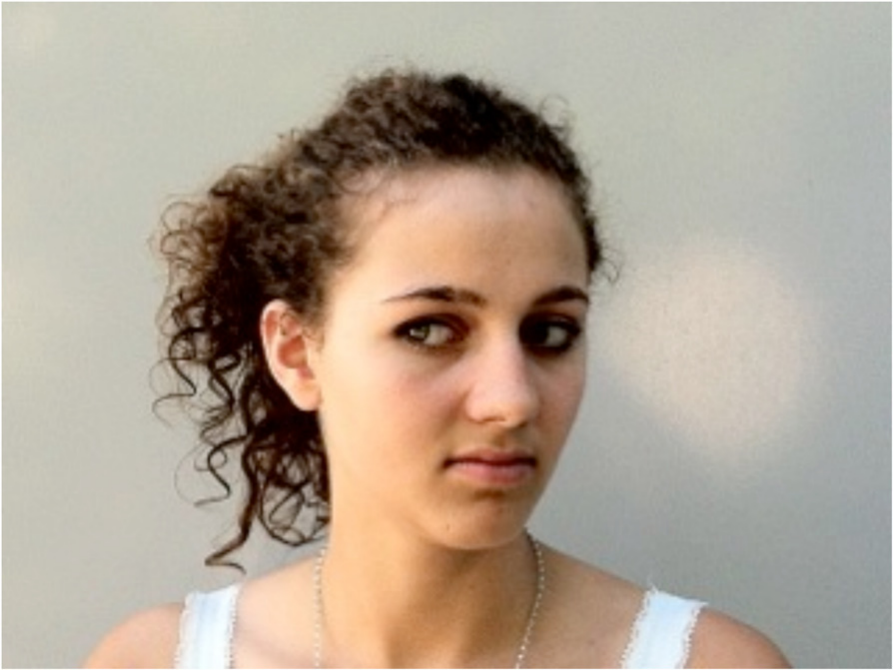
Sequential head and eye movements.

Instruction: "Move your eyes to the right marker while holding your head still. Then, rotate your head to the same marker, keeping your gaze fixed. Thereafter, move your eyes to the left marker while keeping your head on the right side, then rotate your head to the left marker. Do this as precisely and as fast as possible. Continue for 10 seconds" (Figure 5).

#### Eye movements with 45° relative neck rotation to right/left

The eye movements test, as previously described, was repeated with 45° relative neck rotation to the left and right in the sitting and standing positions.

#### Procedure

Test in the sitting position: participants sat on the chair at Position 2 for the left rotation and at Position 3 for the right rotation. They were then asked to turn the head towards the camera, thus creating the 45° relative neck rotation. The markers were adjusted to eye level.

Test in the standing position: participants stood on the footprint with feet together at Position 2 or 3. They were then asked to turn the head towards the camera, thus creating the 45° relative neck rotation. The markers were adjusted to eye level.

Participants were to hold this position and move the gaze between the right and the left markers as fast as possible for 10 seconds.

**Figure 6:**
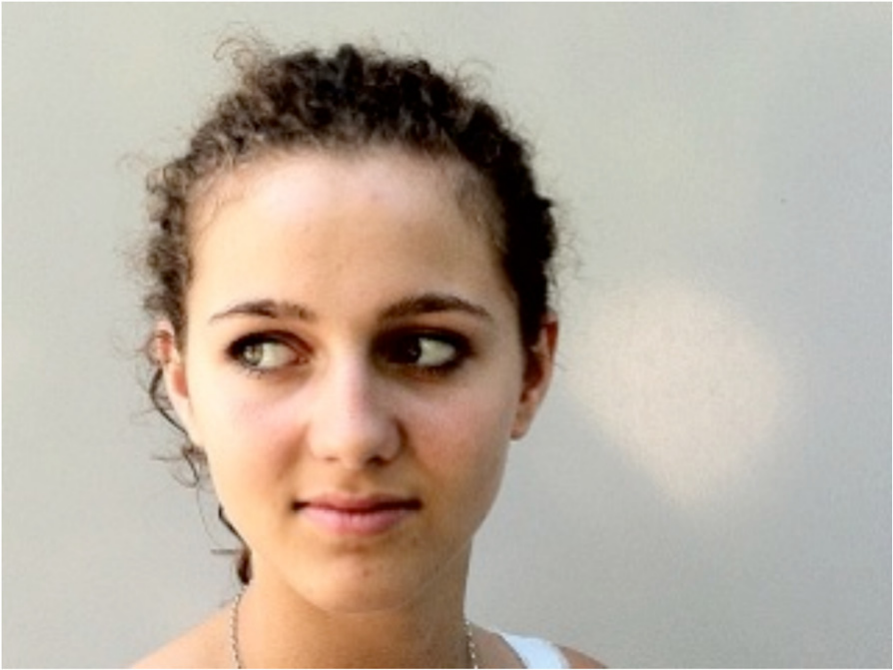
Eye movements with 45° relative neck rotation to the right.

Instruction: "Move your eyes between the two markers as fast as possible, while maintaining your neck in the fixed position". (Figure 6)

The test rating scale was the same as in the previous test for eye movements in the neutral position (Table 2).

Detailed description of the test protocol is available in Additional file 1.

### Test video rating

Randomization: The videos were randomized. Each participant was allocated a number for the whole test battery. These numbers were mixed in a basket and drawn out by an author. Through this method, the order of the videos for the rating process was prearranged.

Two experienced physiotherapists (OMT IFOMPT qualified), blinded to all participants’ data, independently rated the randomized videos. They had had no prior specific training, apart from a few minutes of an instructionvideo. Raters were allowed to view the videos more than once, if necessary. Each test was rated following the criteria described above (Tables 2, 3 and 4).

**Table 4:** Rating scale of sequential head and eye movements

**Rating**	**Definition**
0 = negative	Clear, regular, smooth, dissociated movements of head and eyes.
1 = moderately positive	Slightly decelerated eye movements, occasional associated eye-head movements, head unstable.
2 = strongly positive	Clearly decelerated, irregular and often associated eye-head movements, test not feasible.

### Statistical analysis

Software package R was used for statistical analysis. A weighted Cohen’s kappa (wK) coefficient and confidence interval [95%] was calculated for each test. According to Landis, wK > 0.80 was defined as almost perfect, 0.60 ≤ wK <0.80 as substantial, 0.40 ≤ wK < 0.60 as moderate, 0.21 ≤ wK <0.40 as fair and wK < 0.20 as slight agreement [27]. In this study, acceptable reliability was defined as wK >0.5 and the lower lever of the confidence interval >0.2. In addition to wK the percentage agreement was determined.

The distribution of positive and negative results was calculated for each test. To determine discriminative validity, the number of positive tests (moderate or strong positive) was used as a classifier.

To quantify the performance of the classification, for different cut-off values of the number of positive tests, we calculated the following absolute frequencies: true positives (TP), true negatives (TN), false positives (FP) and false negatives (FN). From these quantities, sensitivity (Sn), specificity (Sp), positive predictive value (PPV) and negative predictive value (NPV) were calculated.

Since a reference test for head-eye movement control impairment is not available, Diagnostic Odds Ratios (DOR) for the different cut-off values were calculated, which defined the associations between ratings and group affiliation. The DOR is the factor by which the chance of impairment is increased with a positive compared to a negative test result. As a global criterion, the Receiver Operating Characteristic (ROC) curve with the Area under the Curve (AUC) was computed. In our context, the AUC is equivalent to the probability that the number of positive tests is larger for impaired than for healthy subjects.

## Results

### Subjects

Patient characteristics (gender, age, NDI score) are described in Table 1. There was no significant difference in age between patients and healthy controls (p-value 0.939).

Included patients had an NDI score of 10 to 62/100, which implies an inhomogeneous representation of symptoms.

### Inter-observer reliability

The results of the inter-observer reliability analysis (weighted kappa coefficient and 95% confidence interval) are presented in Table 5. The weighted kappa coefficient was almost perfect (wK > 0.8) in three tests, substantial (wK 0.69 – 0.79) in five tests and moderate in two tests (wK 0.54 and 0.59). The 95% confidence interval was >0.2 in all tests (0.29 to 0.97). Excellent inter-tester reliability was found for gaze stability and sequential head and eye movements in sitting position, and eye movements in 45° relative neck rotation to the right side in standing position.

**Table 5:** Results inter-observer reliability: weighted kappa (wK) 95% confidence interval (95%CI), percentage agreement (% Agreement)

**Tests: Sitting position**	**wK**	**95% CI**	**% Agreement**
Eye movements	0.72	0.55-0.88	73
Gaze stability	0.86	0.75-0.97	85
Sequential head and eye movement	0.86	0.76-0.97	83
Eye movements in 45° relative neck rotation to the right	0.54	0.29-0.79	69
Eye movements in 45° relative neck rotation to the left	0.79	0.62-0.97	85
**Tests: Standing position**	**wK**	**95% CI**	**% Agreement**
Eye movements	0.78	0.61-0.95	85
Gaze stability	0.59	0.36-0.82	66
Sequential head and eye movement	0.69	0.53-0.85	61
Eye movements in 45° relative neck rotation to the right	0.82	0.67-0.96	85
Eye movements in 45° relative neck rotation to the left	0.70	0.50-0.90	80

### Discriminative validity

On average, three out of five tests in sitting and in standing positions were positive for participants with chronic neck pain. On average, only one test was positive for healthy controls. Results were comparable in the sitting and standing positions.

**Figure 7:**
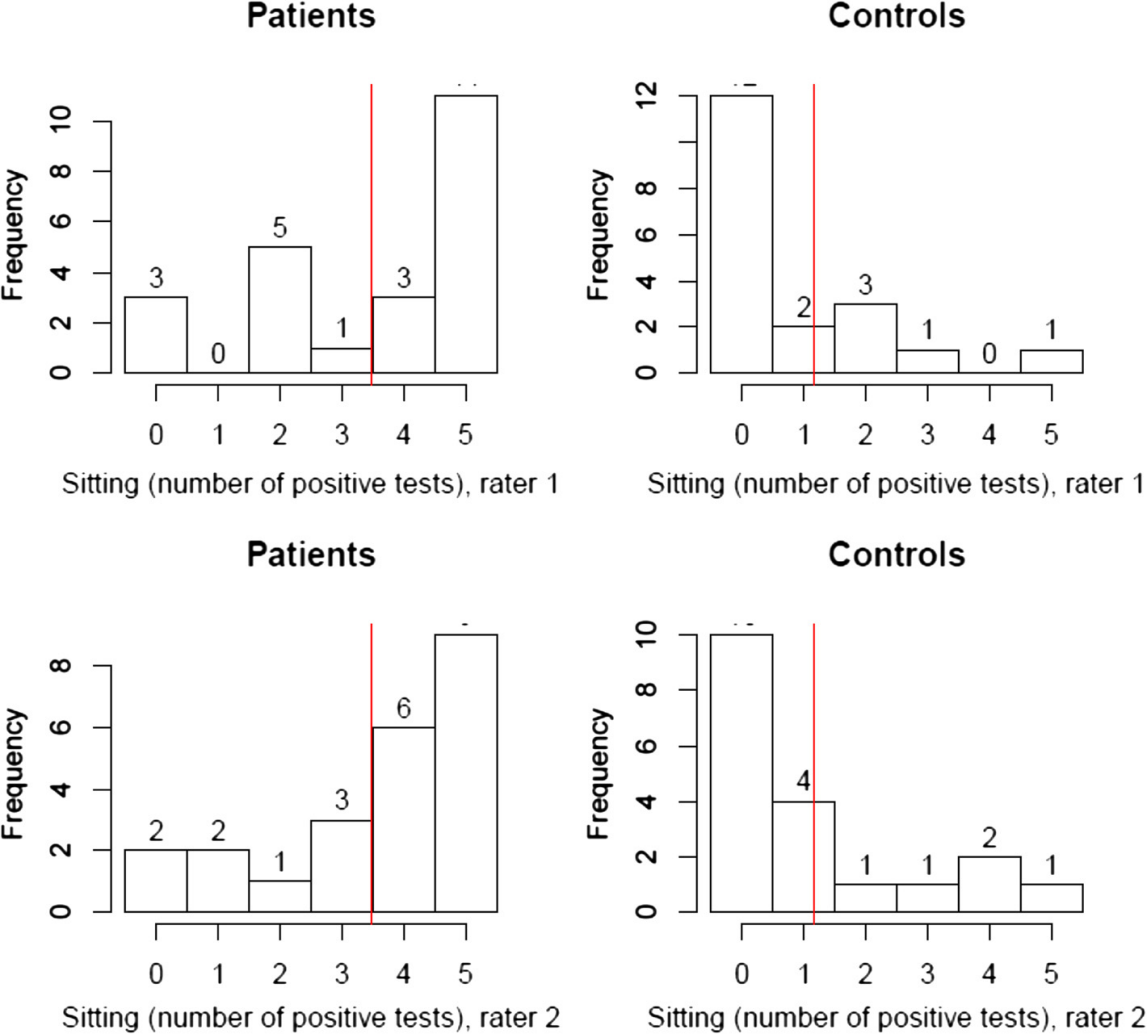
**Distribution of positive tests in sitting.** The red line displays the mean number of positive tests. Results were comparable for Rater 1 and Rater 2.

**Figure 8:**
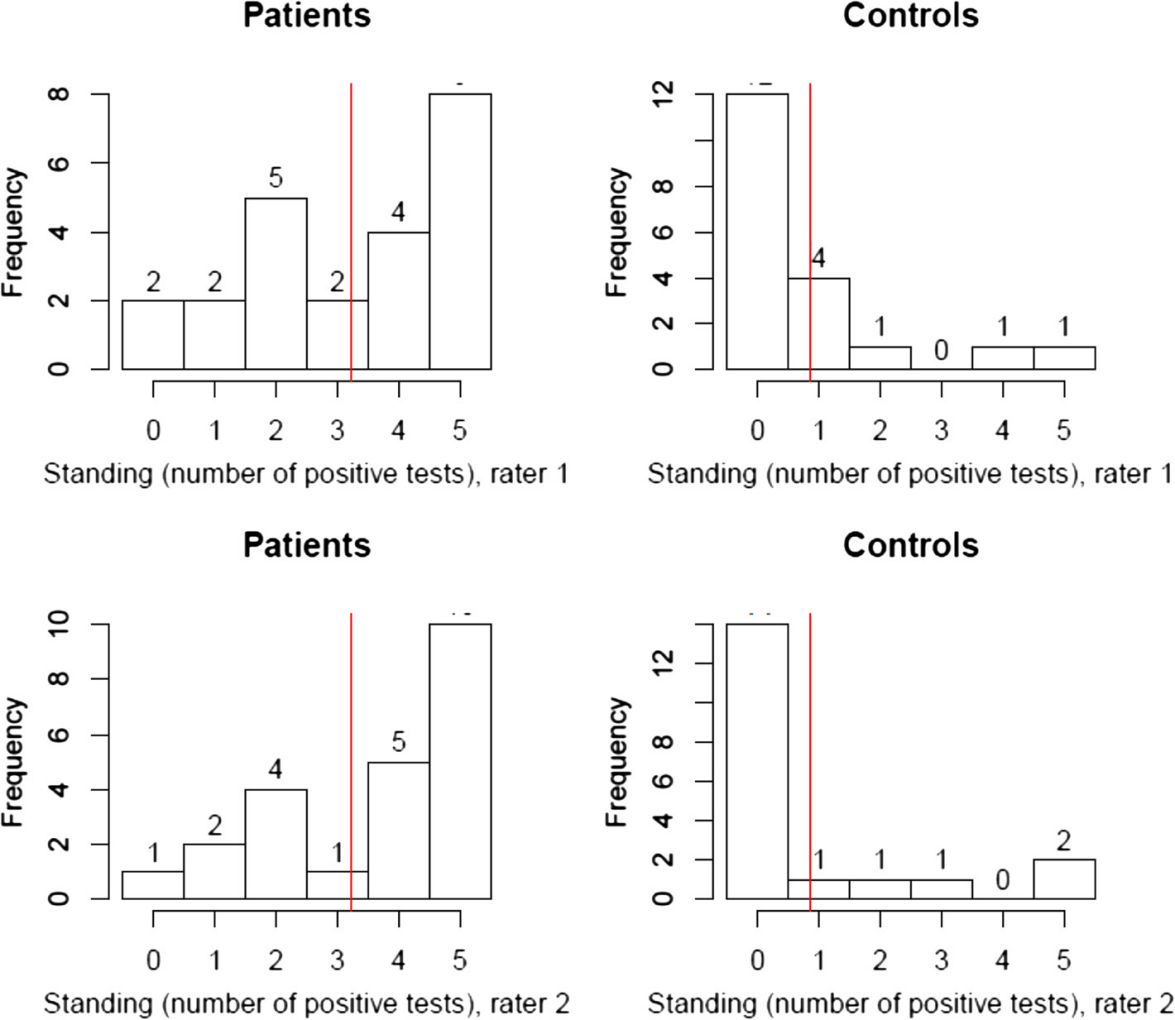
**Distribution of positive tests in standing.** The red line displays the mean number of positive tests. Results were comparable for Rater 1 and Rater 2.

Figures 7 and 8 display the distribution of the numbers of positive tests for the tests in sitting and standing. There was no significant difference between the two positions, or between raters.

**Figure 9:**
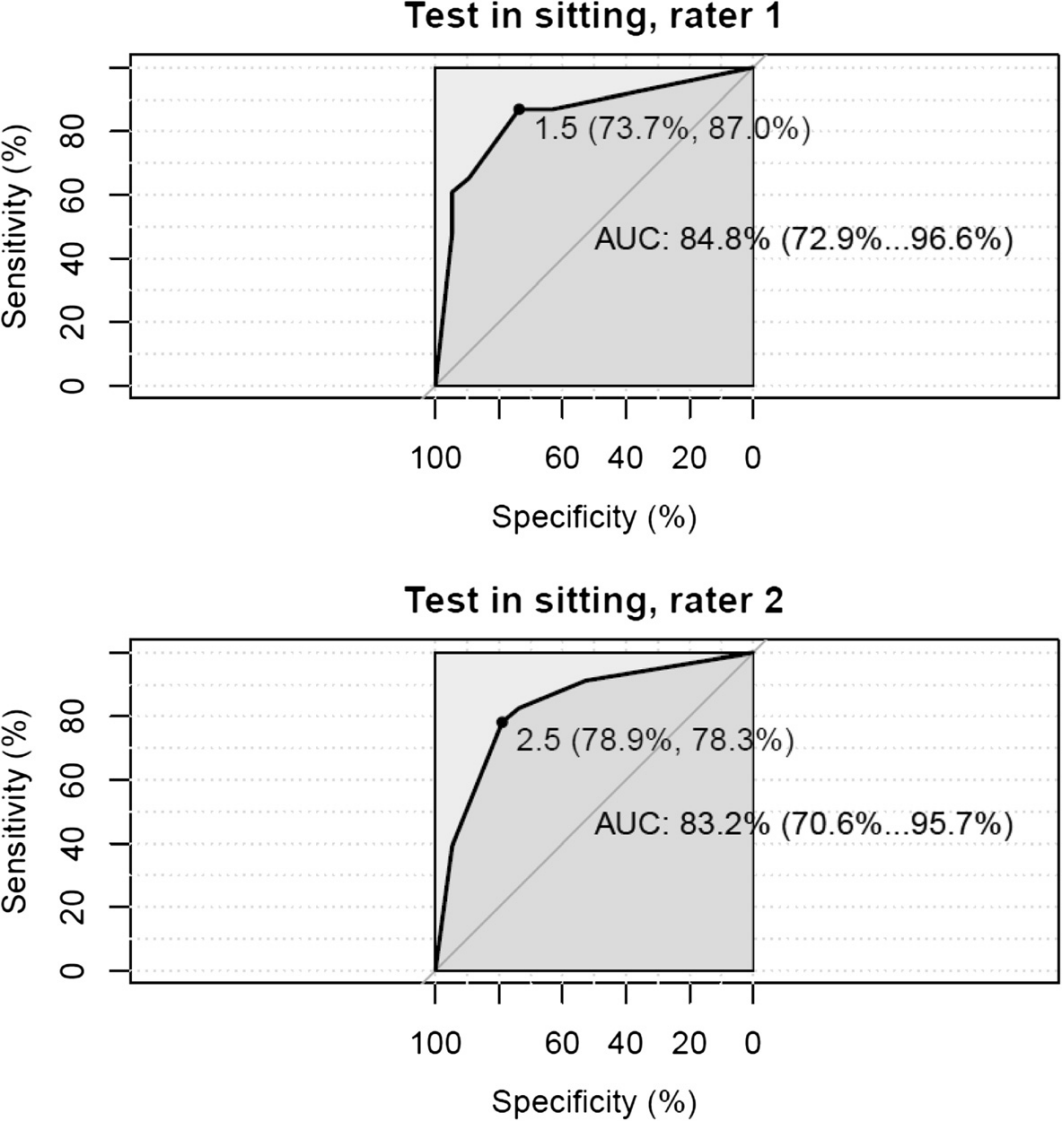
**Receiver operating characteristic (ROC) curves.** Sitting position, Rater 1 and Rater 2 with estimated area under curve AUC (95% CI). The optimal cut-off points for AUC are 1.5 and 2.5.

**Figure 10:**
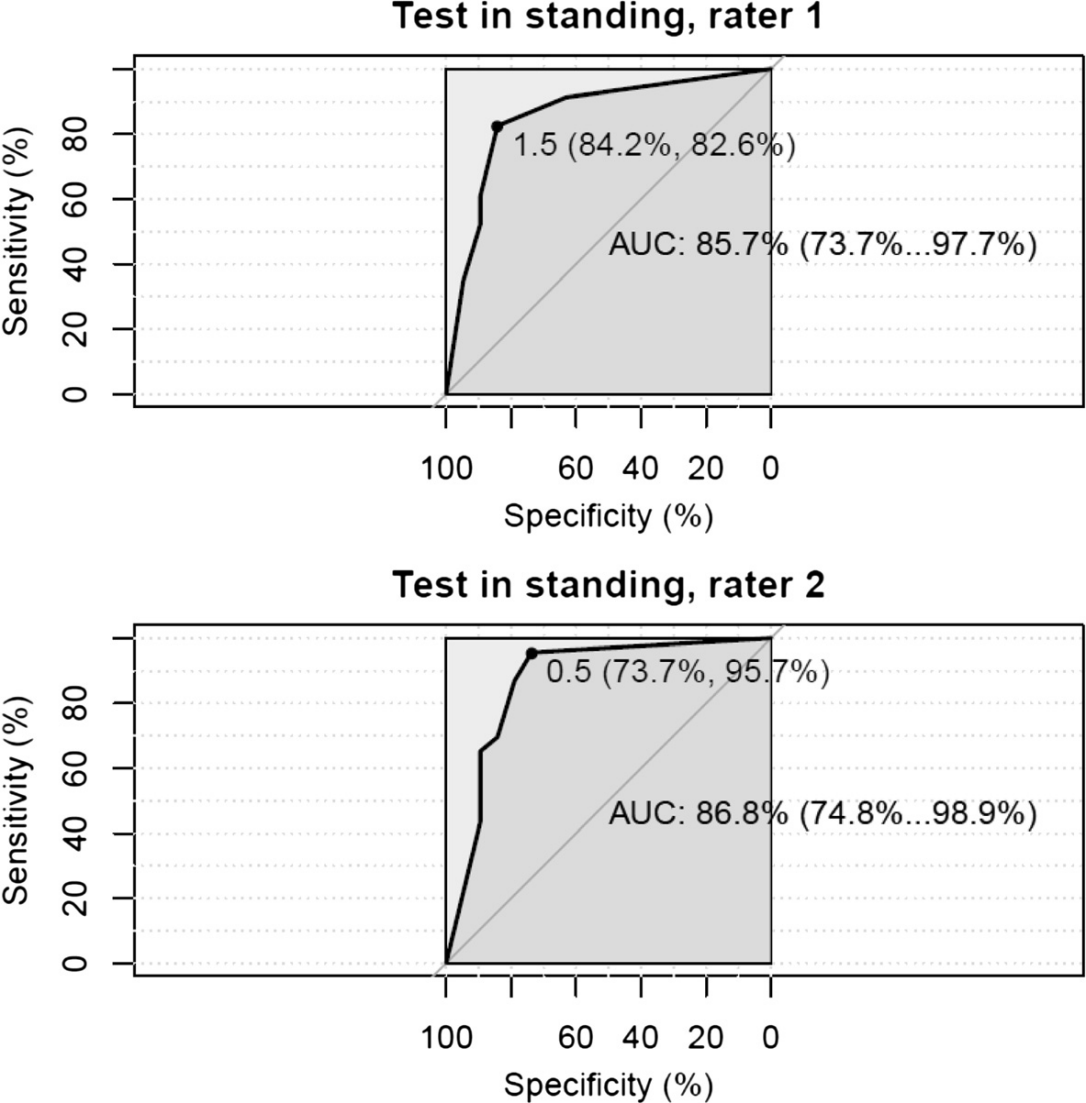
**Receiver operating characteristic (ROC) curves.** Standing position, Rater 1 and Rater 2 with estimated area under curve AUC (95% CI). The optimal cut-off points for AUC are 1.5 and 0.5.

The receiver operating characteristic (ROC) curves with the area under curve AUC (95% CI) show a distribution of the numbers of positive tests in sitting (Figure 9) of 83-85% and in standing (Figure 10) of 85-87%

The Diagnostic Odds Ratio (DOR), test in sitting, of Rater 1 and Rater 2 are 18.6 and 13.3 respectively, using 2 positive tests as the cut-off point.

For the clinical diagnosis of head-eye movement control impairment, a cut-off point of 2 positive ratings out of 5 is recommended. (Tables 6 and 7)

**Table 6:** Results of diagnostic odds ratio DOR by cut-off ≥2/5, ≥3/5, ≥4/5 positive tests

**Rater/ Test**	**Cut-off ≥2/5**	**Cut-off ≥3/5**	**Cut-off ≥4/5**
Rater 1/ Test sitting	18.6	15.9	24
Rater 2/ Test sitting	13.3	13.5	10
Rater 1/ Test standing	25.3	13.2	9.2
Rater 2/ Test standing	25.0	12.2	15.9

**Table 7:** Results of specificity Sp, sensitivity Sn, negative predictive value NPV, positive predictive value PPV Results by cut-off ≥2/5

**Rater/ Test**	**Sp**	**Sn**	**NPV**	**PPV**
Rater 1/ Test sitting	73.68	86.96	82.35	80.00
Rater 2/ Test sitting	73.68	82.61	77.78	79.17
Rater 1/ Test standing	84.21	82.61	80.00	86.36
Rater 2/ Test standing	78.95	86.96	83.33	83.33

## Discussion

Visual rating by physiotherapists of head-eye movement control tests is reliable. Predefined criteria for inter-tester reliability were fulfilled. Similar results for inter-tester reliability were reported by Luomajoki et al. [21], when videos of movement control tests of the lumbar spine were visually evaluated.

In the present study, the discriminative validity was supported with DOR values above 13 to 18, for persons with 2 or more positive tests out of 5. This means that patients with chronic neck pain and associated symptoms, such as dizziness, visual disorders, etc., are up to 13–18 times more likely to have 2 or more tests positive than healthy controls. For the clinical diagnosis of head-eye movement control impairment, a cut-off point of 2 positive results out of 5 tests in sitting or standing position is recommended.

Results showed there was no significant difference between the numbers of positive tests in the sitting and standing positions. Therefore, the hypothesis of an observable increase in head-eye movement control impairment in the standing with feet together position was not confirmed. Since all participants were first tested in sitting and then in standing position, the results also imply that there was no relevant accumulation of fatigue or learning adaptation. The test battery can, therefore, be reduced to five tests. We recommend the tests to be performed in the sitting position in order to minimise the risk of falling in patients with dizziness.

Visible differences between controls and patients were identified in the tests performed: patients with chronic neck pain showed a decelerated velocity of head and eye movements and distinctive movement quality. These results are in line with previous research [3].

The strength of these tests is that they can be performed by all subjects and do not require technical equipment, such as an oculograph. Results showed that quality differences were visually identifiable during the10 seconds performance per test. This is important for general clinical use, since assessment is fast and requires little installation. These tests could be of assistance in the daily clinical practice of physiotherapists, in deciding whether rehabilitation for eye-head movement control impairment is necessary.

Based on the results from previous research, methodological adaptations to the tests were made in order to adapt them to a clinical environment. The measurement of quantities, such as velocity and range of motion, is impossible without technical equipment; therefore, our tests were rated on the quality of movements. In the present study, impaired quality of movement control was characterized by a short interruption during the change of direction and irregular or uncoordinated, decelerated eye movements. Decreased active stabilization of the head position during the eye movements test was also a positive rating criterion. Difficulty in keeping a fixed gaze during the gaze stability test was a regular occurrence in the patient group and was a criterion for a positive rating. Difficulty in dissociating head and eye movements in the sequential test was typical and clearly visible for a positive rating in this test.

The rating of the head-eye movement control tests used in this study can be easily learned from a short instruction video.

The methodology had to be adapted so that the tests could be videotaped. The chosen videotaping method meant that we were only able to measure horizontal eye movement. Other movement directions would have resulted in the pupil being covering by the eye-lid on the tape. This limitation would not be present in daily clinical practice, where vertical and diagonal movements could also be assessed".

To exclude the impact of pain avoidance strategies [28] and mimic reactions from the videos, an inclusion criterion was a symptom-free active range of 45° head rotation to both sides.

There are certain limitations in the present study that need to be addressed. The intake of drugs was not considered. Since medication may have an influence on the velocity of head-eye movements, this information should be provided. The incidence of light was not standardized but, as there was no direct light from any side, it did not disturb the participants.

Contrary to clinical practice, raters were allowed to watch the videos several times. This may have improved inter-observer reliability. In clinical practice however, the physiotherapist is also able to repeat testing and, in addition, use symptom provocations and other assessments for clinical diagnostics. Unexpectedly, no male patients were included in the study because of recruitment problems. However, neck pain and WAD are more common in women [2,29].

Contrary to Treleaven and colleagues [3], the study also included patients with no neck acceleration trauma. The reason for including these subjects was that research has shown that head-eye coordination impairment is also prevalent in neck pain patients without trauma [29,30]. Two participants of the controls reported minor historical neck trauma but, since they had had no neck pain problems for many years, they were also included in the control group.

Further research should evaluate test-retest reliability in patients with stable symptoms. A correlation between head-eye coordination and NDI scores was not evaluated in this study, nor were differences in the performance of the trauma and non-trauma patient groups. These could be of further interest.

Moreover, following diagnosis and treatment, changes in head-eye coordination tests should be evaluated in relation to changes in patients’ limitations in daily life.

## Conclusion

Visual assessment by physiotherapists of head-eye movement control tests is reliable. No differences between test performance in the sitting and standing positions were identified: the test battery can be reduced to five tests. Our subsequent recommendation was that tests be performed in the sitting position. The test battery can discriminate between patients with chronic neck pain and healthy controls. Two or more positive tests out of five can be interpreted as impaired head-eye movement control. Further research is needed to determine test-retest stability and responsiveness.

## Competing interests

The authors declare that they have no competing interests.

## Authors' contributions

EDC, JAH, JK and HL designed the study. EDC and JAH collected subjects’ data. AM performed statistical analysis. All authors were involved in writing the manuscript. All authors read and approved the final manuscript.

## Authors' information

EDC: physiotherapist FH MAS ZHAW, PT OMT IFOMT, CRAFTA®, owner of a physiotherapy practice in the -Zentrum für Manuelle Therapie- Zurich, instructor at the University of Zurich, Department of Chiropractic Medicine, CH-8008 Zurich. JAH: physiotherapist FH MAS ZHAW, PT, OMT IFOMT, owner of a physiotherapy practice in CH-8132 Egg, instructor Kaltenborn- Evjenth-Conzept®. AM: physiotherapist, MSc Statistics, Institute of Physiotherapy, School of Health Professions, ZHAW Zurich University of Applied Sciences, CH-8401 Winterthur. HL: physiotherapist, MSc, PhD, Researcher and Lecturer, Institute of Physiotherapy, School of Health Professions, ZHAW Zurich University of Applied Sciences, CH-8401 Winterthur. JK: physiotherapist, MSc, PhD, Head of Research and Development, Institute of Physiotherapy, School of Health Professions, ZHAW Zurich University of Applied Sciences, CH-8401 Winterthur.

## Pre-publication history

The pre-publication history for this paper can be accessed here:

http://www.biomedcentral.com/1471-2474/15/16/prepub

## Supplementary Material

Additional file 1 Test description and instructions. Click here for file
