# Supplementary material for: Head-Eye movement control tests in patients with chronic neck pain; Inter-observer reliability and discriminative validity
Source: BMC Musculoskelet Disord. 2014 Jan 14;15:16. doi: 10.1186/1471-2474-15-16 (PMC3893395; doi:10.1186/1471-2474-15-16)
Supplement: Additional file 1 — Test description and instructions. [file 1471-2474-15-16-S1.docx]

Additional file 1

Test description and instructions

| Test | Description | Instruction |
| --- | --- | --- |
| Eye movements Sitting | Free upright sitting. Eye movements to the right and left side to the marker maintaining the head in neutral position | Maintaining the head in neutral position while moving your eyes between the two marker |
| Gaze stability Sitting | Free upright sitting. Gaze stable to the camera while moving the head left and right | Gaze stable to the camera while moving the head left and right |
| Sequential head and eye movements Sitting | Free upright sitting. Sequential head and eye movements with eye movements to the right while head remains stable, followed by head rotation to the right, then repeat of procedure changing direction to the left | Move your eyes to the right marker while the head remains stable and then rotate your head to the right. Next move your eyes to the left marker and then rotate your head to the left. |
| Eye movements with 45° rotation position of the cervical spine to the right Sitting | Free upright sitting at Position 2. Head turned towards to the camera. This induces the cervical rotation to the right. Eye movements to the right and left side markers, maintaining the head in rotated position | Maintain the rotated position of your head while moving your eyes between the two markers |
| Eye movements with 45° rotation position of the cervical spine to the left Sitting | Free upright sitting at Position 3. Head turned towards to the camera. This induces the cervical rotation to the right. Eye movements to the right and left side markers, maintaining the head in rotated position | Maintain the rotated position of your head while moving your eyes between the two markers |
| Eye movements Standing | Upright standing with feet together. Eye movements to the right and left side markers, maintaining the head in neutral position | Maintain your head in neutral position while moving your eyes between the two markers |
| Sequential head and eye movements Standing | Upright standing with feet together. Sequential head and eye movements with eye movements to the right while the head remains stable, thereafter the head rotates to the right; then repeat the procedure changing direction to the left | Move your eyes to the right marker while the head remains stable and then follow your head in rotation to the right. Move your eyes then to the left marker and then rotate your head to the left. |
| Eye movements with 45° rotation position of the cervical spine to the right Standing | Upright standing with feet together at Position 2. Head turned towards the camera. This induces the cervical rotation to the right. Eye movements to the right and left side markers, maintaining the head in rotated position | Maintain the rotated position of your head while moving your eyes between the two markers |
| Eye movements with 45° rotation position of the cervical spine to the left Standing | Upright standing with feet together at Position 3. Head turned towards the camera. This induces the cervical rotation to the left. Eye movements to the right and left side markers, maintaining the head in rotated position. | Maintain the rotated position of your head while moving your eyes between the two markers |
